# Elevated vitreous Lipocalin-2 levels of patients with proliferative diabetic retinopathy

**DOI:** 10.1186/s12886-020-01462-5

**Published:** 2020-06-30

**Authors:** Hui Wang, Hui Lou, Yongrong Li, Fengtao Ji, Wei Chen, Qianyi Lu, Guoxu Xu

**Affiliations:** 1 Department of Ophthalmology, The Second Affiliated Hospital of Soochow University, 1055 Sanxiang Road, Suzhou, 215004 China; 2 Department of Ophthalmology, The Second People’s Hospital of Hefei, Hefei, 230011 China; 3 Department of Ophthalmology, Affiliated Hospital of Nantong University, Nantong, 226001 Jiangsu China; 4 Department of Ophthalmology, The First Affiliated Hospital of Soochow University, Suzhou, 215006 China

**Keywords:** Diabetic retinopathy, Vascular endothelial growth factor, Lipocalin-2

## Abstract

**Background:**

Lipocalin-2 (LCN2) is a novel adipokine with potential roles in obesity, insulin resistance, and inflammation. This study aims to assess the concentrations of LCN2 and vascular endothelial growth factor (VEGF) expressed in the vitreous humors of patients with proliferative diabetic retinopathy (PDR).

**Methods:**

The concentrations of LCN2 and VEGF were measured from the vitreous of 67 patients undergoing vitrectomy (20 controls and 47 PDR) via enzyme-linked immunosorbent assay (ELISA). Patients with non-ocular pathology that could elevate the LCN2 level in the vitreous were excluded. PDR activity and a history of panretinal photocoagulation were used for further grouping analysis.

**Results:**

The vitreous concentration of LCN2 was statistically significantly higher in the PDR group compared to the control group (63,522 (30,009) pg/ml versus 1663 (1191) pg/ml, respectively; *P* < 0.001). VEGF level was also significantly higher in the PDR group than in the control group (1038 (1326) pg/ml versus 9 pg/ml, respectively; *P* < 0.001). The mean vitreous LCN2 and VEGF levels in active PDR patients were significantly higher than that of the inactive PDR patients. The mean LCN2 concentration in vitreous humor was significantly lower in the 28 PDR patients with a history of complete PRP (37,304 (16,651) pg/mL) in comparison with 19 PDR patients without preperformed panretinal photocoagulation or with preperformed incomplete panretinal photocoagulation (79,796 (24,391) pg/mL). A significant correlation between the vitreous LCN2 level and VEGF level was found in patients with PDR (R = 0.34; *P* = 0.019).

**Conclusions:**

This report shows a significant increase of LCN2 in the vitreous fluid of patients with PDR and present a significant correlation between LCN2 and VEGF, suggesting LCN2 might be involved in the pathogenesis of PDR.

## Background

Diabetic retinopathy (DR) is a serious sight-threatening complication of diabetes. In the USA, studies estimate that 28.5–40.3% of patients with type 2 diabetes had DR, and 4.4–8.2% of DR is vision threatening [1]. In a Japanese study, the incidence of DR was 26.6% in patients with type 2 diabetes who did not have DR at baseline over 8 years, and 15.0% of mild non-proliferative DR showed progression to proliferative DR (PDR) [2].

Chronic low-grade subclinical inflammation in the state of persistent hyperglycemia has an effect on retinal microvascular and eventually leads to DR. [3] Aberrant neovascularization following retinal ischemia is a clinical feature of PDR, which can lead to vitreous hemorrhage and tractional retinal detachment, thus affecting vision [4]. Several studies have indicated that inflammatory mediators were involved in DR, and inflammatory cells produced angiogenic cytokines and growth factors, which were implicated in the progression of DR [5–7].

Lipocalin-2 (LCN2), also known as neutrophil gelatinase-associated lipocalin (NGAL), is released by various cell types that has been found to be involved in multiple processes such as metabolic homeostasis, apoptosis, infection, immune response, or inflammation [8]. In the existing literature, elevated LCN2 was also detected in the aqueous humor of patients with idiopathic acute anterior uveitis [9] and central retinal vein occlusion [10] and vitreous fluid from patients with rhegmatogenous retinal detachment [11]. A large number of studies indicate that the level of LCN2 is closely related to the degree of inflammation in various inflammatory diseases [12–17]. However, LCN2 level in vitreous fluid and the relation with other inflammatory cytokines such as vascular endothelial growth factor (VEGF) in diabetic patients with DR or PDR remain unclear. Therefore, the present study was aimed to investigate a possible correlation between LCN2 and VEGF levels in the vitreous of eyes with PDR to provide supporting evidence of LCN2’s potential involvement in the progress of PDR and investigate the potential ability of LCN2 as biomarker of PDR.

## Methods

### Study population

This study enrolled 67 patients: 47 diabetic patients with PDR had vitrectomies for vitreous hemorrhage or tractional retinal detachment and a control group of 20 non-diabetic patients had vitrectomies for idiopathic macular holes, only one eye from each patient being included. Patients with non-ocular pathology that could elevate the LCN2 level in the vitreous such as cardiovascular diseases, intestinal inflammation, breast cancer, pancreatic diseases, etc. were excluded. Samples from eyes obtained during a repeat vitrectomy were excluded. Table 1 presents the backgrounds and demographics of the 67 patients. This study was conducted in accordance with the Declaration of Helsinki and written informed consent was obtained from all patients. The study was approved by the ethical committee of The Second Affiliated Hospital of Soochow University.

**Table 1:** Intravitreous Concentrations of LCN2 and VEGF in the PDR and Control Groups

	PDR Group	Control Group	*P*
No. of cases	47	20	
Gender (male: female)	16:31	7:13	0.82
Age (years)	61.32 (7.22)	59.85 (6.90)	0.38
HbAlc (%)	8.16 (2.44)	5.03 (0.51)	< 0.001
VH	32	–	
TRD	6	–	
VH + TRD	9	–	
Idiopathic macular hole	–	20	
LCN2 (pg/ml)	63,522 (30,009)	1663 (1191)	< 0.001
VEGF (pg/ml)	1038 (1326)	<9^a^	< 0.001

*LCN2* Lipocalin-2, *VEGF* vascular endothelial growth factor; *PDR* proliferative diabetic retinopathy, *HbAlc* glycosylated hemoglobin, *VH* vitreous hemorrhage, *TRD* tractional retinal detachment

^a^Vitreous level of VEGF regarded as 9 pg/mL in control participants

PDR was classified as active and inactive. Inactive PDR was defined as the presence of nonvascularized fibrotic epiretinal membranes, and was present in 15 patients. Active PDR was defined as visible large new vessels within the proliferative tissue, and was present in 32 patients. The clinical information of patients’ eyes was recorded during the operation, and the operative videotapes were kept for subsequent analysis. These data were reviewed by three ophthalmologists independently to avoid a bias before further testing of LCN2 and VEGF concentrations in vitreous fluid.

### Sample collection

Vitrectomy was performed using a standard three-port 25-gauge PPV technique. Undiluted vitreous samples (0.6 to 1.0 ml) were aspirated from the mid vitreous at the initial stage of vitrectomy, and were placed on ice in sterile tubes (Eppendorf) immediately. The tubes were then centrifuged at 13,000 rpm for 5 min at 4 °C within 30 min. The supernatant was dispensed as 200 μL aliquots and stored at − 80 °C until analysis was performed. Enzyme-linked immunosorbent assay (ELISA) was used to determine the concentration of LCN2 or VEGF in the samples.

### Measurement of LCN2 and VEGF

LCN2 levels were analyzed using a commercially available ELISA kit (BioVendor, Brno, Czech Republic) according to the manufacturer’s instructions. Recombinant LCN2 was serially diluted to obtain a standard graph by a standard method. The analyses were made in duplicate for each sample. The same method was used to detect VEGF in vitreous fluid with ELISA kit of human VEGF (R&D Systems, Minneapolis, Minnesota, USA).

### Statistical analysis

SPSS 17.0 software (SPSS Inc., Chicago, IL, USA) was used to perform the statistical analyses. Data are expressed as means (standard deviation). For quantitative variables, differences between two groups were determined by Student’s t test (2-tailed). The primary outcome of this study was to compare the vitreous concentrations of LCN2 and VEGF of PDR patients with non-diabetic patients. The secondary outcomes were the comparison of LCN2 and VEGF concentrations between inactive PDR and active PDR, and the comparison between eyes with and without complete panretinal photocoagulation (PRP). Correlation between the concentration of vitreous LCN2 and VEGF was quantified using the Pearson correlation coefficient. The differences were considered significant at *P* < 0.05.

## Results

Table 1 summarizes the surgical indications of PDR patients, including 32 eyes of vitreous hemorrhage, 6 eyes of tractional retinal detachment, and 9 eyes of vitreous hemorrhage with tractional retinal detachment. Hemoglobin in vitreous had a significant effect on the results. To prevent this, patients with recent vitreous hemorrhage (< 1 month) were not enrolled in this study.

**Fig. 1:**
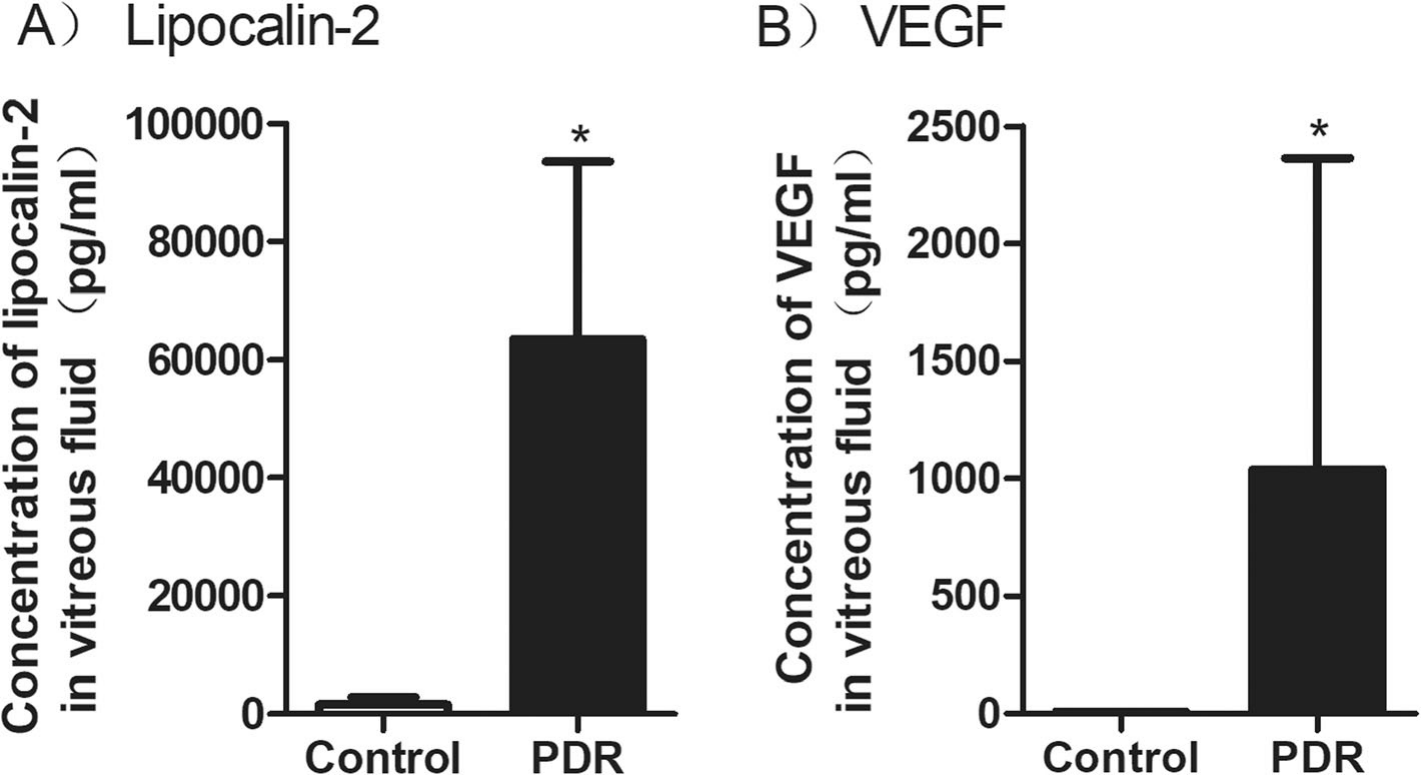
**a** Vitreous LCN2 levels in eyes with nondiabetic control group and eyes with PDR. **b** Vitreous vascular endothelial growth factor (VEGF) level in eyes nondiabetic control group and eyes with PDR. PDR = proliferative diabetic retinopathy. * *P* < .0001

The vitreous levels of LCN2 were significantly higher in the PDR group than in the control group (63,522 (30,009) pg/ml versus 1663 (1191) pg/ml, respectively; *P* < 0.001) (Fig. 1a). VEGF levels were also significantly higher in the PDR group than in the control group (1038 (1326) pg/ml versus 9 pg/ml, respectively; *P* < 0.001) (Fig. 1b). In fact, VEGF was not detected in patients with idiopathic macular hole, and 9 pg/ml represents the sensitivity limit of the detection kit.

**Fig. 2:**
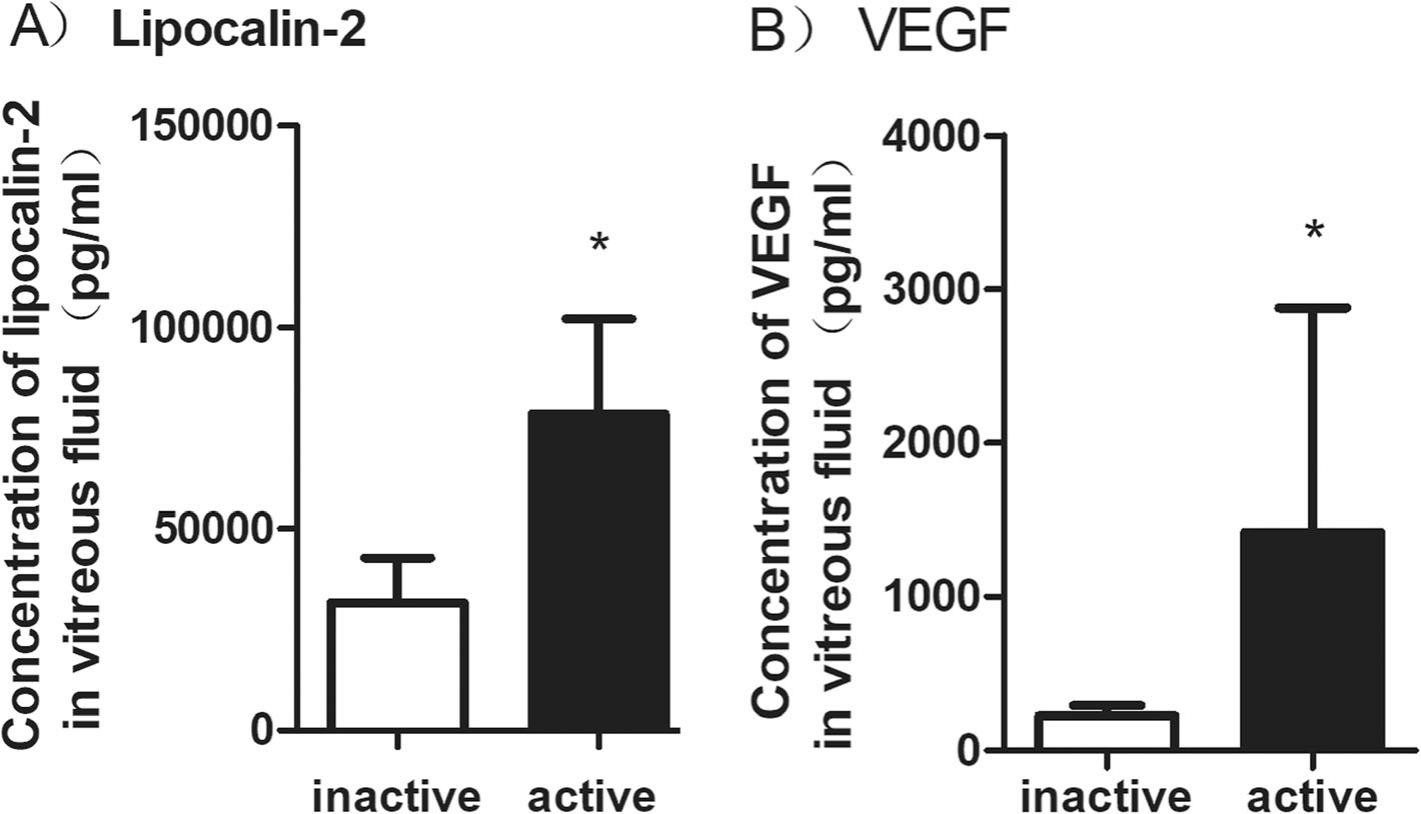
**a** Lipocalin-2 levels in vitreous samples of active and inactive PDR. **b** Vascular endothelial growth factor (VEGF) levels in vitreous samples active and inactive PDR. PDR = proliferative diabetic retinopathy. *P < .0001

**Fig. 3:**
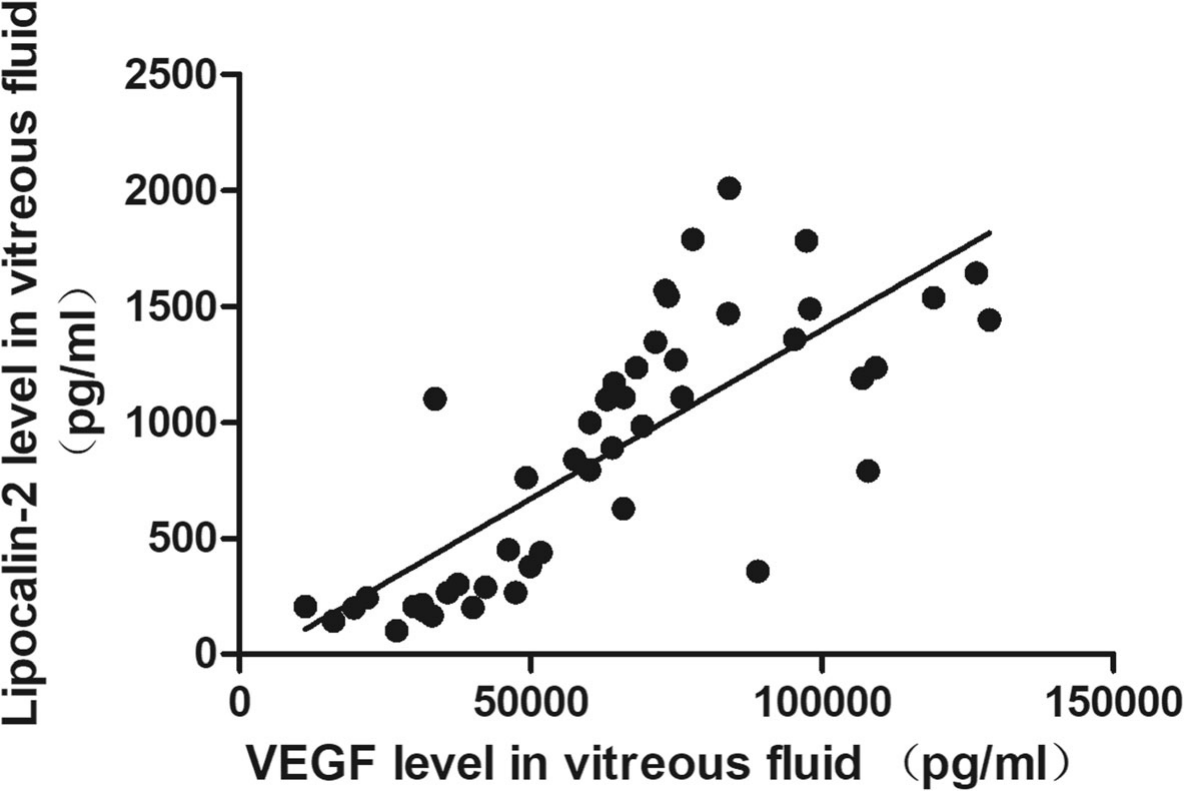
A linear relationship between Lipocalin-2 and vascular endothelial growth factor (VEGF) in vitreous fluid with proliferative diabetic retinopathy. [Pearson correlation coefficient] R = 0.34; *P* = 0.019

We further divided PDR patients (47 eyes) into active PDR (32 eyes) and inactive PDR (15 eyes). The mean vitreous level of LCN2 was 78,465 (23,637) pg/ml in eyes of patients with active PDR and 31,645 (11,122) pg/ml in eyes of patients with inactive PDR. The mean vitreous LCN2 level in active PDR patients was significantly higher than that of the inactive PDR patients (*P* < 0.001; Fig. 2a). The mean vitreous level of VEGF was 1419.2 (1461) pg/ml in the eyes with active PDR and 224 (69) pg/ml in the eyes with inactive PDR. This difference was statistically significant (*P* < 0.001, Fig. 2b). Our findings also demonstrated a significant correlation between the vitreous LCN2 level and VEGF level in patients with PDR (R = 0.34; *P* = 0.019; Fig. 3).

**Fig. 4:**
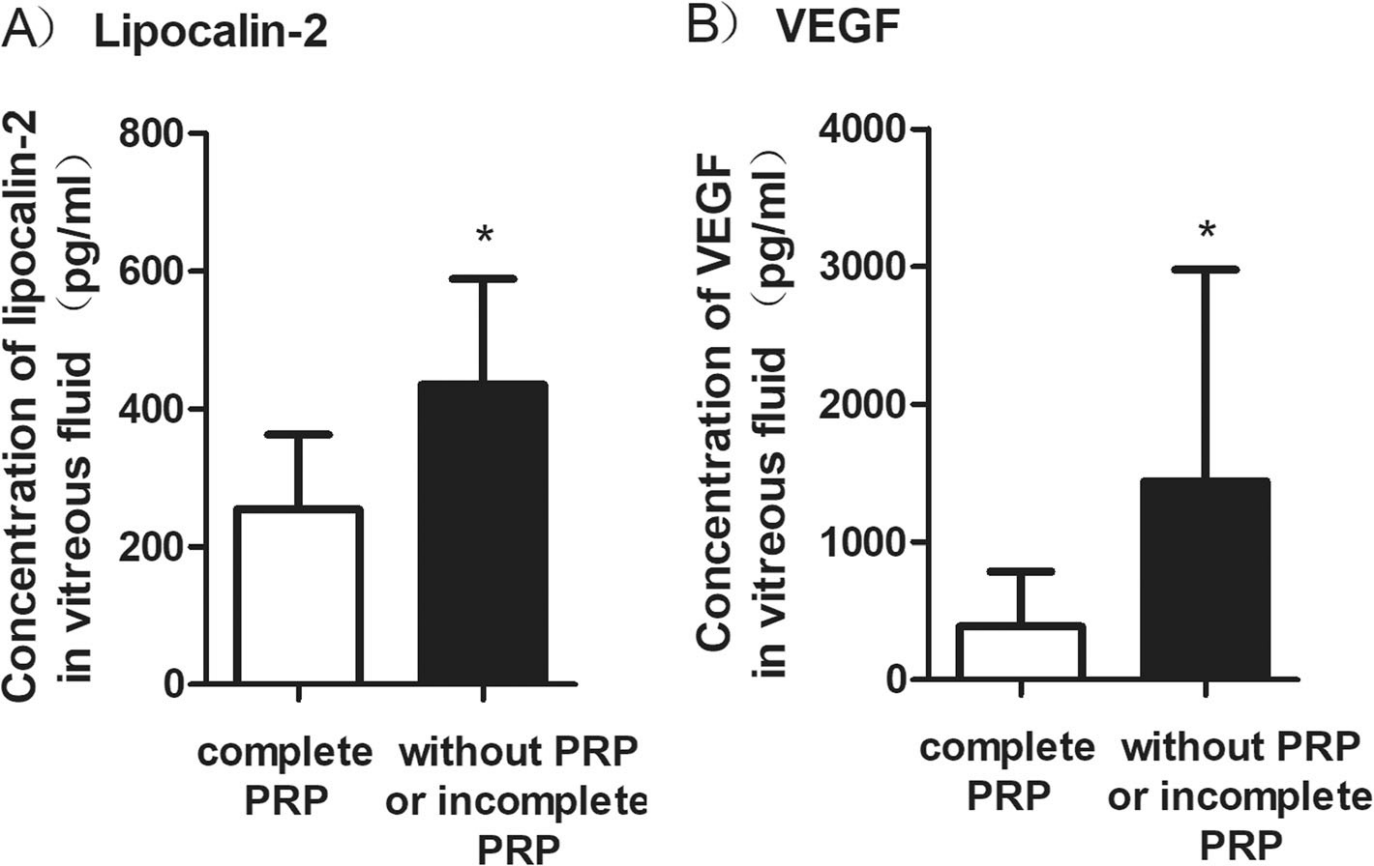
**a** Vitreous LCN2 levels in 28 PDR eyes with preperformed complete PRP and 19 PDR eyes without preperformed PRP or with preperformed incomplete PRP. **b** Vascular endothelial growth factor (VEGF) levels in 28 PDR eyes with preperformed complete PRP and 19 PDR eyes without preperformed PRP or with preperformed incomplete PRP. PDR = proliferative diabetic retinopathy; PRP = panretinal photocoagulation. *P < .0001

The effect of PRP on the vitreous levels of LCN2 and VEGF was also evaluated in our study. Of 47 patients with PDR, 28 (57.4%) were performed with complete PRP (1500 to 3000 spots of burns) 17.2 (20.1) months (2–46) before vitrectomy. The mean LCN2 concentration in vitreous humor was lower in the 28 PDR patients with a history of complete PRP (37,304 (16,651) pg/mL) in comparison with 19 PDR patients without preperformed PRP or with preperformed incomplete PRP (79,796 (24,391) pg/mL) (Fig. 4a). This difference was considered statistically significant (*P* < 0.001). We also analyzed the relationship between the duration from PRP to vitrectomy and the concentration of LCN2 and VEGF in the vitreous of all 28 PDR patients preperformed with complete PRP. Pearson’s correlation analysis indicated that there was no significant correlation between the duration from PRP to vitrectomy and the vitreous level of LCN2 or VEGF (*P* = 0.139 and *P* = 0.205, respectively).

## Discussion

The results indicated that vitreous LCN2 and VEGF levels were significantly higher in the PDR group compared to the nondiabetic control group. To our knowledge, this is the first study in the literature evaluating vitreous LCN2 in patients with PDR. Although whether PDR in this study was in active phase depended on our clinical experience, there might be some mistakes, but it does not affect our conclusion that vitreous LCN2 in active PDR was significantly higher than that in inactive PDR. These findings suggest that LCN2 may be involved in the vascularization of PDR.

LCN2 has been identified as a potential biomarker for several common inflammatory diseases including acute kidney injury, lupus nephritis, cardiovascular diseases, or intestinal inflammation [18]. Although LCN2 is highly upregulated under a large number of inflammatory conditions, both pro- and anti-inflammatory properties of this adipokine have been reported. Hu et al. described that LCN2 upregulation protects hepatocytes from interleukin-1beta-induced inflammation [19]. Vichaya found that LCN2 is dispensable for sterile inflammation-induced sickness and depression-like behavior [16]. Recently, LCN2 was also found to be involved in the pathogenesis of Alzheimer’s disease [20]. In terms of ophthalmic diseases, the work of Parmar et al. have revealed that LCN2 plays a regulatory role in retinal inflammation during retinal degeneration such as Stargardt, retinitis pigmentosa and age-related macular degeneration. They found that LCN2 induced expression of antioxidant enzymes heme oxygenase 1 and superoxide dismutase 2 in RPE cells and could inhibit the cytotoxic effects of H_2_O_2_ and LPS, and exacerbated inflammation following light exposure was observed in LCN2 knockout mice model of Stargardt disease and age-related macular degeneration [21]. In the study of the Yaran Koba group, they demonstrated that increased LCN2 levels in the eyes of patients with central retinal vein occlusion begin an intense neuroinflammatory process and cause iron accumulation in retinal cells which may lead to the complications such as macular edema, macular ischemia, and neovascularization [10]. In a recent study, it has been demonstrated that a positive correlation between vitreous levels of LCN2 and proliferative vitreoretinopathy grading in patients with rhegmatogenous retinal detachment, revealing a potential role in the pathogenesis and progression of proliferative vitreoretinopathy. They thought LCN2 seems to play a role in reactive gliosis and neuroinflammation, which are involved in PVR pathophysiology.

At present, VEGF is recognized as an important protein in the pathogenesis of PDR, and anti-VEGF therapy has achieved good results in clinical use. Not surprisingly, we also found a significant increase in intravitreal VEGF concentration in PDR patients. The mean concentration of vitreous VEGF in eyes with PDR in our study was similar with that in other studies [22, 23]. Previous studies have found that successful PRP can effectively reduce the concentration of VEGF in the vitreous. Our study found that the concentrations of LCN2 and VEGF in vitreous fluid of PDR patients who had completed PRP were significantly lower than those of PDR patients who had not completed PRP. This indicates that PRP might affects the production of LCN2 and VEGF simultaneously. Similar results were obtained when comparing the concentrations of LCN2 and VEGF in vitreous fluid of inactive PDR with active PDR. This suggests that both LCN2 and VEGF may be related to the activity of neovascularization. The exact mechanisms of PRP are unclear, but it is possible that the decreased area of retinal tissue leads to improved oxygenation and a reduction in the levels of VEGF. A reduction in levels of VEGF may be important in reducing the risk of harmful new vessels forming. In our study, PRP reduced LCN2 concentration in vitreous cavity, the reason may be that PRP destroyed retinal tissues and reduced LCN2 secretion.

The significant correlation between the vitreous LCN2 and VEGF levels in eyes with PDR suggests that there may be upstream-downstream relationship between the two proteins. Yang et al. previously reported that VEGF was significantly increased with LCN2 expression in MCF-7 human breast cancer cells [24]. They found LCN2-induced VEGF was mediated through hypoxia-inducible factor 1alpha (HIF-1α). Intravitreous VEGF and HIF-1α in diabetic patients with PDR were found increased and related mutually previously [25]. Moreover, the production of VEGF was diminished in a diabetic mice model lacking HIF-1α expression [26]. These findings clearly support the hypothesis that LCN2 upregulates VEGF expression through HIF-1α and promotes neovascularization in PDR.

The limitation of this study is that the number of eyes in the study group and the control group was unbalanced at baseline, but the statistical value *P* in our primary outcome was less than 0.001, which does not seem to affect our conclusion that the concentration of LCN2 and VEGF in the vitreous cavity of PDR patients is higher than that of the control group.

## Conclusions

In conclusion, this study provides evidence that the levels of both LCN2 and VEGF are elevated in the vitreous fluid from patients with PDR compared to non diabetic patients. Although our study demonstrates a significant correlation between LCN2 and VEGF levels, it does not establish a definitive cause and effect relationship between LCN2 and VEGF, and further mechanism research will be needed. Our findings indicate the potential role of LCN2 in the development and progression of PDR. Further studies will be required to confirm the possible molecular mechanisms of LCN2 that contribute to inflammatory response and retinal angiogenesis in animal models of DR, which will help to determine the precise role of LCN2 as a potential PDR biomarker or therapeutic target.

## Data Availability

The datasets used and analysed in the current study are available from the corresponding author on reasonable request.

## Abbreviations

LCN2: lipocalin-2
VEGF: vascular endothelial growth factor
PDR: proliferative diabetic retinopathy
ELISA: enzyme-linked immunosorbent assay
HbAlc: glycosylated hemoglobin
VH: vitreous hemorrhage
TRD: tractional retinal detachment
PRP: panretinal photocoagulation
